# Supplementary material for: In Vitro Evaluation of Anti-Rift Valley Fever Virus, Antioxidant and Anti-Inflammatory Activity of South African Medicinal Plant Extracts
Source: Viruses. 2021 Jan 31;13(2):221. doi: 10.3390/v13020221 (PMC7912315; doi:10.3390/v13020221)
Supplement: Supplementary file 1 [file viruses-13-00221-s001.pdf]

viruses

Supplementary Information

In Vitro Evaluation of Anti-RIFT Valley Fever Virus, Antioxidant and Anti-Inflammatory Activity of South African Medicinal Plant Extracts

Garland K. More [1,*], Raymond T. Makola [2,3] and Gerhard Prinsloo, [1]

[1] College of Agriculture and Environmental Sciences, University of South Africa, Private Bag X6, Florida 1710, South Africa; moregk@unisa.ac.za (G.K.M.); prinsg@unisa.ac.za (G.P.)
[2] Department of Biochemistry Microbiology and Biotechnology, School of Molecular and Life Science, University of Limpopo (Turfloop Campus) Sovenga 0727; makolaraymond4@gmail.com (R.T.M.)
[3] National institution of communicable diseases, Special Viral Pathogen/ Arbovirus Unit, 1 Modderfontein Rd, Sandringham, Johannesburg 2192
* Correspondence: moregk@unisa.ac.za

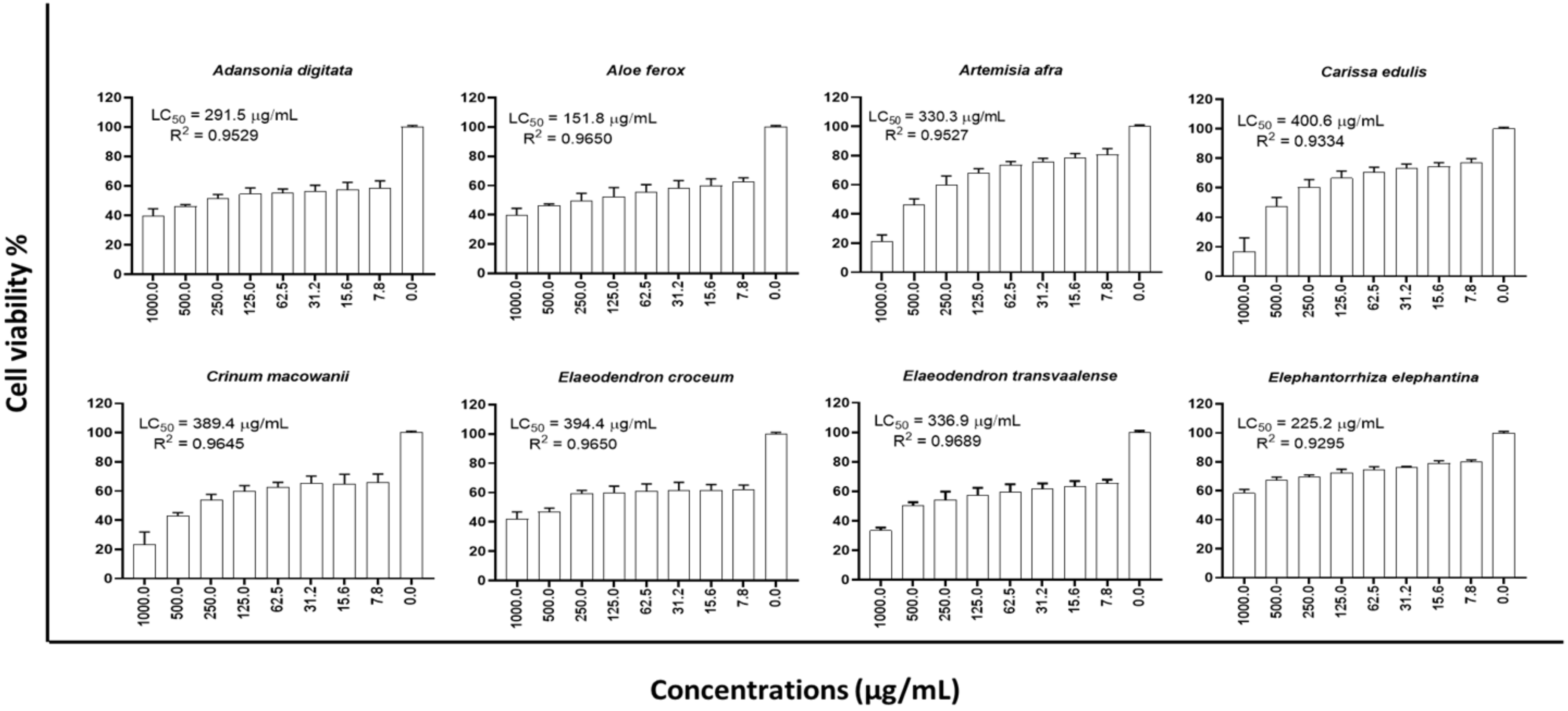
Cell viability %
Adansonia digitata
LC50 = 291.5 μg/mL
R2 = 0.9529
Aloe ferox
LC50 = 151.8 μg/mL
R2 = 0.9650
Artemisia afra
LC50 = 330.3 μg/mL
R2 = 0.9527
Carissa edulis
LC50 = 400.6 μg/mL
R2 = 0.9334
Crinum macowanii
LC50 = 389.4 μg/mL
R2 = 0.9645
Elaeodendron croceum
LC50 = 394.4 μg/mL
R2 = 0.9650
Elaeodendron transvaalense
LC50 = 336.9 μg/mL
R2 = 0.9689
Elephantorrhiza elephantina
LC50 = 225.2 μg/mL
R2 = 0.9295
0 20 40 60 80 100 120
1000.0 500.0 250.0 125.0 62.5 31.2 15.6 7.8 0.0
Concentrations (μg/mL)

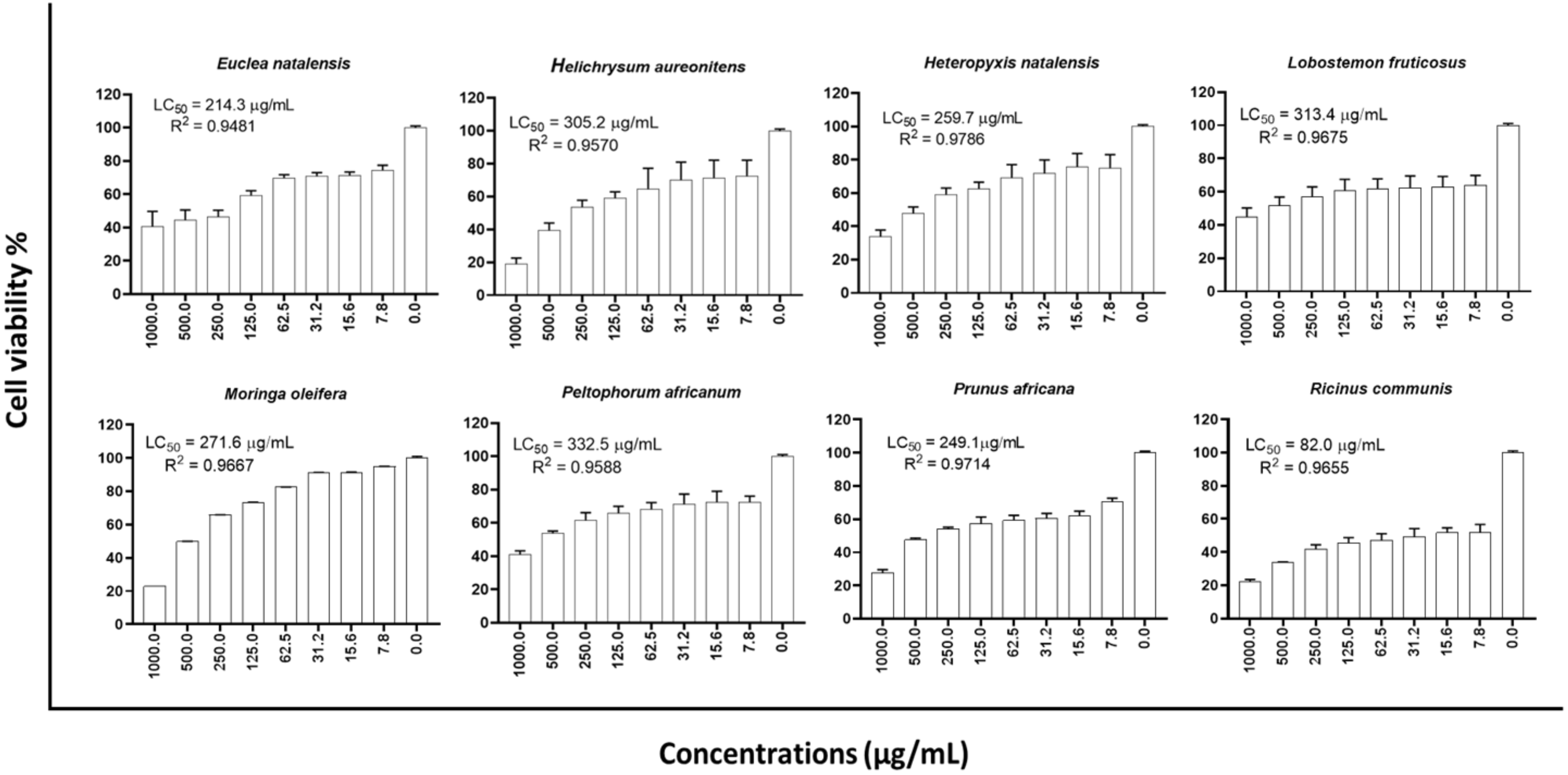
Cell viability %
Euclea natalensis
LC50 = 214.3 μg/mL
R2 = 0.9481
Helichrysum aureonitens
LC50 = 305.2 μg/mL
R2 = 0.9570
Heteropyxis natalensis
LC50 = 259.7 μg/mL
R2 = 0.9786
Lobostemon fruticosus
LC50 = 313.4 μg/mL
R2 = 0.9675
Moringa oleifera
LC50 = 271.6 μg/mL
R2 = 0.9667
Peltophorum africanum
LC50 = 332.5 μg/mL
R2 = 0.9588
Prunus africana
LC50 = 249.1μg/mL
R2 = 0.9714
Ricinus communis
LC50 = 82.0 μg/mL
R2 = 0.9655
1000.0 500.0 250.0 125.0 62.5 31.2 15.6 7.8 0.0
Concentrations (μg/mL)

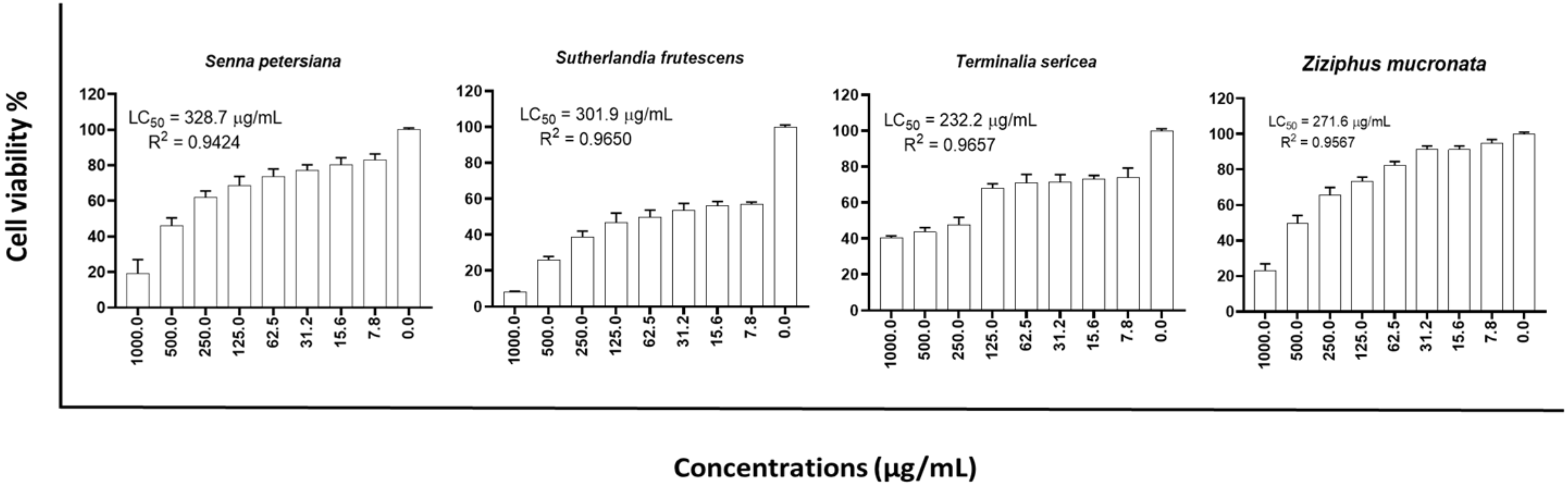

Figure S1: Lethal concentration (LC50 = μg/mL) of the MTT assays following 48 hours treatment with twenty plant extracts well known for their antiviral activity.

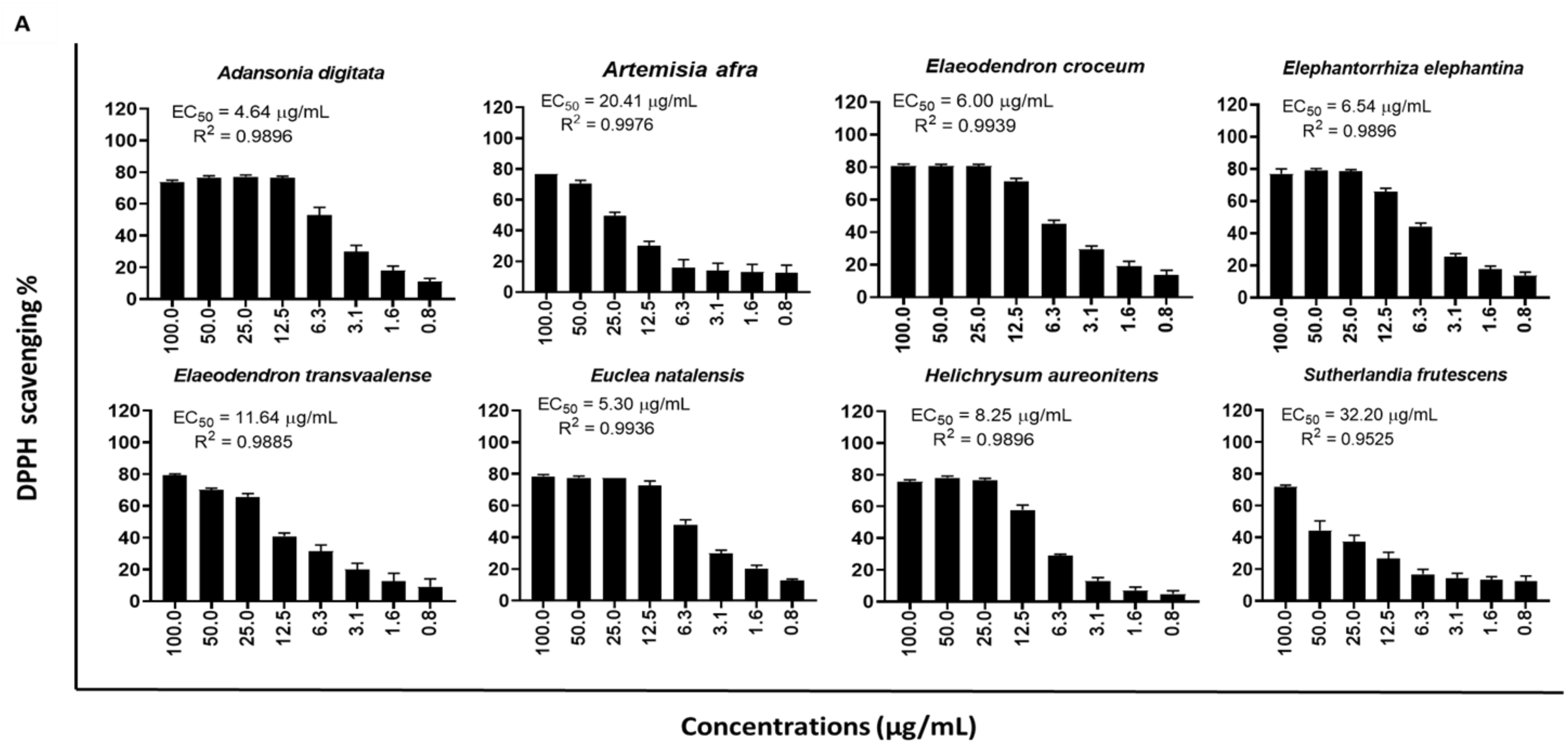
A
DPPH scavenging %
Adansonia digitata
EC50 = 4.64 µg/mL
R2 = 0.9896
Artemisia afra
EC50 = 20.41 µg/mL
R2 = 0.9976
Elaeodendron croceum
EC50 = 6.00 µg/mL
R2 = 0.9939
Elephantorrhiza elephantina
EC50 = 6.54 µg/mL
R2 = 0.9896
Elaeodendron transvaalense
EC50 = 11.64 µg/mL
R2 = 0.9885
Euclea natalensis
EC50 = 5.30 µg/mL
R2 = 0.9936
Helichrysum aureonitens
EC50 = 8.25 µg/mL
R2 = 0.9896
Sutherlandia frutescens
EC50 = 32.20 µg/mL
R2 = 0.9525
0 20 40 60 80 100 120
100.0 50.0 25.0 12.5 6.3 3.1 1.6 0.8
Concentrations (µg/mL)

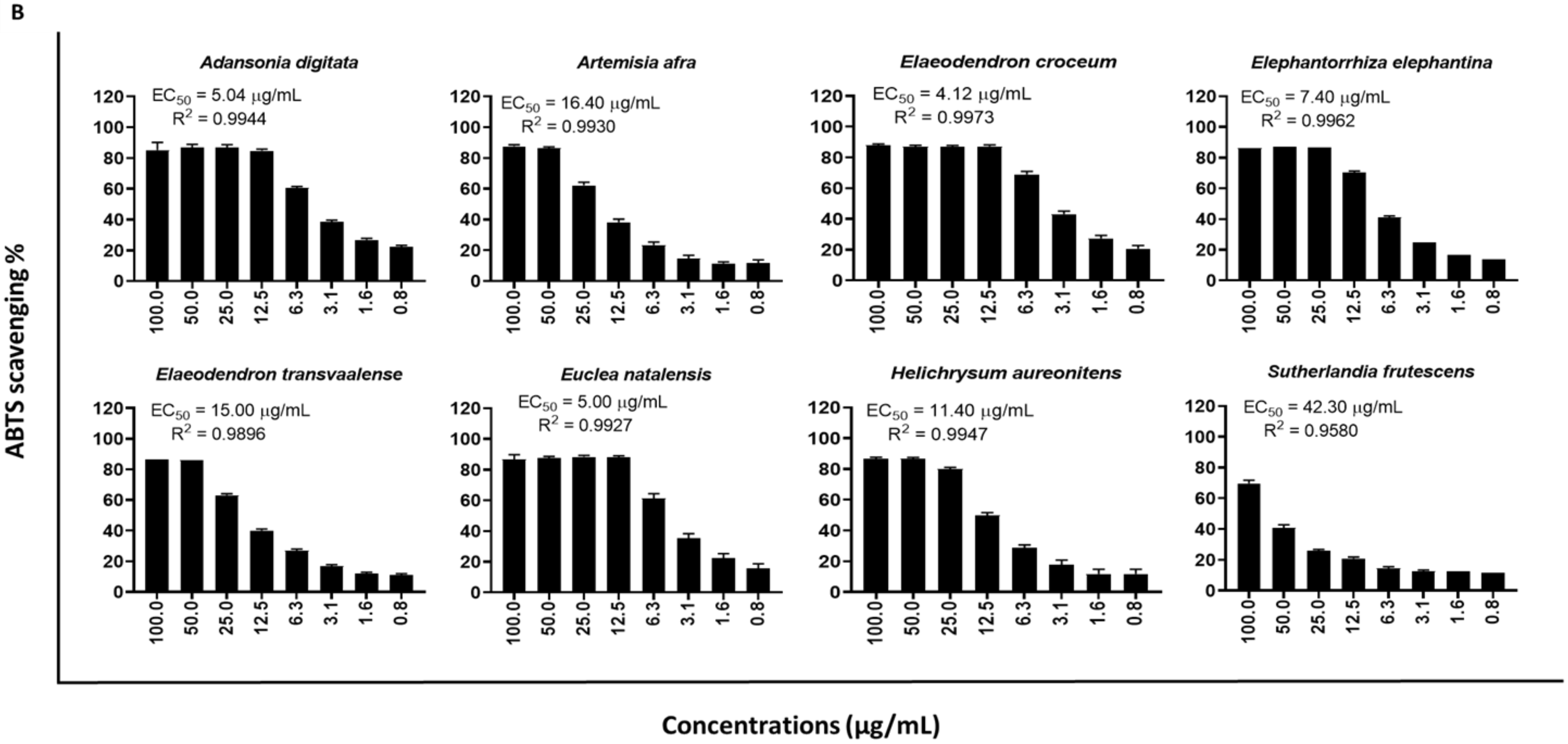

Figure S2: Effective concentration (EC_{50} = µg/mL) of the DPPH **(A)** and ABTS **(B)** assays following 48 hours treatment with eight plant extracts well known for their antiviral activity.
